# Turmeric Oil Interferes with Quorum Sensing as an Alternative Approach to Control *Aeromonas hydrophila* Infection in Aquaculture

**DOI:** 10.3390/biology14050483

**Published:** 2025-04-27

**Authors:** Jing Dong, Jian Tong, Shengping Li, Xinwei Ma, Qiuhong Yang, Yongtao Liu, Shun Zhou, Xizhi Shi, Xiaohui Ai

**Affiliations:** 1 Yangtze River Fisheries Research Institute, Chinese Academy of Fishery Sciences, Wuhan 430223, China; 2 College of Veterinary Medicine, Hunan Agricultural University, Changsha 410125, China; 3 Key Laboratory of Aquacultural Biotechnology, Ministry of Education, Ningbo University, Ningbo 315211, China

**Keywords:** turmeric oil, aquaculture, *Aeromonas hydrophila*, quorum sensing, anti-infective

## Abstract

Aquatic products play a crucial role in meeting the increasing global demands for high-quality proteins. However, the occurrence of bacterial diseases results in significant economic losses worldwide. *Aeromonas hydrophila* (*A. hydrophila*) is the pathogen of several fish diseases. Antibiotics were widely used in combating bacterial diseases in aquaculture. The increasing occurrences of antibiotic resistance necessitate the restricted use of antibiotics. Consequently, developing drugs that avoid antibiotic resistance is important for the future of aquaculture. Quorum sensing (QS) is critical for bacterial pathogens in regulating bacterial virulence and is a promising target for developing anti-infective agents. Here, we found that turmeric oil with a MIC of 256 μg/mL could dose-dependently reduce the virulence phenotypes regulated by QS, ranging from 8 to 64 μg/mL, suggesting that sub-inhibitory concentrations of turmeric oil could inhibit bacterial virulence. Further qPCR findings demonstrated that turmeric oil could significantly inhibit the transcription of *aerA*, *ahyI*, and *ahyR* by a 54-fold, 36-fold, and 56-fold change reduction, respectively. Cell live/dead staining and animal study results showed that turmeric oil could inhibit the pathogenicity of *A. hydrophila*. Fish treated with turmeric oil showed a reduced mortality rate of 60%, whereas all fish in the positive control group died. Moreover, treatment with turmeric oil could alleviate the renal injury. Collectively, the results suggested that targeting bacterial virulence might be a useful approach to combating bacterial infections, and turmeric oil could serve as a potential agent for combating *A. hydrophila* infections.

## Simple Summary

*Aeromonas hydrophila* is a bacterial pathogen that causes severe economic losses to the industry. The occurrence of antibiotic resistance has limited the effectiveness of antibiotics. Therefore, screening drugs combating *A. hydrophila* infections is essential for the healthy development of aquaculture. Here, turmeric oil was screened as an anti-infective agent targeting the quorum sensing of *A. hydrophila*. Experimental therapeutics demonstrated that the mortality of fish after turmeric oil treatment showed a significant decrease post *A. hydrophila* infection. Compared with antibiotics, turmeric oil offers less selective pressure to the bacteria. The findings provided an alternative strategy for developing drugs against bacterial infection and a promising agent for combating *A. hydrophila*-associated diseases.

## 1. Introduction

*Aeromonas hydrophila* (*A. hydrophila*) is an opportunistic pathogen that can cause a number of diseases in aquatic animals [1,2,3]. Therefore, *A. hydrophila* brings millions of dollars in economic losses to the aquaculture industry every year [4]. Moreover, *A. hydrophila* is regarded as a bacterial pathogen that can cause infections in humans and animals [1]. Antibiotics are the major route for treating bacterial diseases in aquaculture; however, the selective pressure caused by antibiotics results in antibiotic resistance and decreases the therapeutic effect [5]. Meanwhile, the accumulation of antibiotics in humans through the food chain poses serious health risks [6]. Thus, it is reasonable to develop novel drugs based on alternative strategies against *A. hydrophila*-associated diseases in aquaculture.

*A. hydrophila* can produce several virulence factors that contribute to pathogenicity, including hemolysin, aerolysin, and heat-stable enterotoxin [7]. Several of these virulence factors are recognized as potent drug-screening targets. Bacterial chemical communication mediated by quorum sensing (QS) using autoinducers is essential for bacteria producing virulence factors, which contributes to the pathogenicity [8]. Therefore, disrupting QS rather than directly killing bacterial cells or inhibiting bacterial growth has been identified as an effective approach to controlling bacterial infections.

Herbal medicines with multi-biological activities have been widely used in dealing with diseases for centuries. Moreover, natural compounds isolated from herbal medicines and other natural sources provide active molecules for developing drugs for clinical use [9]. Turmeric (*Curcuma longa* L.), a perennial herb native to Asia, has been used as a medicine, dye, cosmetic, and food seasoning [10]. As a medicine, turmeric is widely used for dealing with a number of diseases, including hepatic and gastrointestinal disorders, fever, inflammation, and even sepsis [11,12]. Turmeric oil, isolated from turmeric rhizomes, is composed of various sesquiterpenes [13,14]. Turmeric oil has several biological functions, such as antioxidant, antibacterial, antifungal, anti-tumor, and anti-inflammatory activities [15,16,17,18]. To overcome antibiotic resistance caused by antibiotics, natural antibacterial alternatives have become an attractive approach to managing bacterial infections [19]. It is known that essential oils isolated from herbal medicines are rich in bioactive compositions. Turmeric oil has been reported to enhance immunity in combating *A. hydrophila* infection in aquaculture [20,21]. However, the anti-infective activity of turmeric oil against *A. hydrophila* is not well understood. This study aimed to clarify the influence of turmeric oil at sub-inhibitory concentrations on QS-regulated virulence expression and to provide a clue in developing anti-infective drugs based on the anti-virulence strategy.

## 2. Materials and Methods

### 2.1. Microorganisms and Reagents

*A. hydrophila* XS-91-4-1 was a clinical strain isolated from a silver carp that suffered from bacterial hemorrhagic septicemia, and *Chromobacterium violaceum* CV026 was a mutant strain without N-acyl homoserine lactones (AHLs). Turmeric oil with a purity of 80% was a commercial product obtained from Shanghai Yuanye Biotechnology Co., Ltd. (Shanghai, China). Turmeric oil was prepared in DMSO to a stock solution of 40,960 μg/mL for in vitro assays, while a turmeric oil emulsion was obtained using 10% Tween-80 for animal studies.

### 2.2. GC-MS Analysis of Turmeric Oil

The chemical contents were determined by GC-MS on an Agilent 7890B-6977A (Santa Clara, CA, USA) The oven was set to 40 °C and kept for 3 min. Samples were injected on a VF-5MS column with 30 m in length, 0.25 mm of internal diameter, and 0.25 μm of film thickness. The temperature was programmed as follows: an initial temperature of 40 °C was maintained for 3 min, then it was switched to 260 °C at a rate of 20 °C/min and maintained for 11 min. The injection temperature was set to 250 °C, and helium was chosen as the carrier gas at 1 mL/min. Turmeric oil without any dilution was injected at a volume of 0.2 μL. Scan mode was set up ranging from 12 to 550 amu.

### 2.3. Minimum Inhibitory Concentration (MIC)

The MIC assay was carried out according to methods supplied by CLSI [22]. Briefly, turmeric oil was diluted 2-fold from 1024 μg/mL to 2 μg/mL in a 96-well plate at a volume of 100 μL. Then, 100 μL of bacterial cells at a final concentration of 5 × 10^5^ cfu/mL were mixed with the drugs in each well, and the plate was incubated at 28 °C overnight. The impact of the solvent was measured by the addition of DMSO to the turmeric oil-free wells. The results were read by the unaided eye, and wells with no visible growth were defined as MIC.

### 2.4. Growth Curves

We added 1 mL of overnight bacterial suspension to 100 mL fresh LB medium and then cultured at 28 °C until the bacterial density (OD) at 600 nm reached 0.3 (early logarithmic phase). Then, the inoculum was divided into 5 flasks, each containing 15 mL, and turmeric oil was added to achieve final concentrations of 0, 8, 16, 32, and 64 μg/mL, respectively. The bacteria were further cultured at 28 °C for 5 h, and the absorptions at 600 nm were determined every 30 min by a visible spectrometer. DMSO with the same volume as 64 μg/mL turmeric oil group was added to the turmeric oil-free group to assess the impact of DMSO.

### 2.5. Hemolysis Assay

Hemolysis mediated by aerolysin in cell-free supernatants was measured according to methods described previously [23]. Briefly, turmeric oil at the indicated concentrations was added to the bacterial cultures when the OD_600nm_ reached 0.3, and then the inocula were cultured in a shaker to an OD_600nm_ of 1.5. Centrifugation was performed to obtain cell-free bacterial supernatants from bacterial suspensions with different concentrations of turmeric oil. Aerolysin in bacterial supernatants was first activated by trypsin. Then, 100 μL of activated bacterial supernatants were used for the hemolysis assay. The hemolytic reactions were carried out in 1.5 mL tubes containing 100 μL bacterial supernatant, 875 μL hemolysis buffer, and 25 μL sheep erythrocytes. After incubation at 37 °C for 20 min, the values of OD_543nm_ were measured. Cells treated with 0.1% Triton X-100 (Yuanye, Shanghai, China) were defined as 100% hemolytic activity.

### 2.6. Immunoblotting

Immunoblotting was conducted by Dong et al. [24]. Briefly, a BCA protein assay kit (Thermo Fisher Scientific, Waltham, MA, USA) was used first to quantify the amounts of proteins in the cell-free supernatants described above. Then, samples were prepared by adding the Laemmli sample buffer to the supernatants and were loaded onto a sodium dodecyl sulfate (SDS)-polyacrylamide (12%) gel for electrophoresis after boiling. A semi-dry blotter was used to transfer the proteins from the gel onto a PVDF membrane. The membrane was then incubated with 5% non-fat milk for 2 h, followed by incubation with a primary anti-aerolysin antibody and an HRP-conjugated secondary goat anti-rabbit antiserum for 1 h. The proteins were determined using ECL detection reagents.

### 2.7. Lipase Assay

The amount of lipase after treatment with turmeric oil was evaluated according to the protocol reported by Srinivasan et al. [25]. The reaction mixtures contained 100 μL of bacterial supernatant and 900 μL of substrate mixture. The substrate mixture was composed of 0.3% (*w*/*v*) p-nitrophenyl palmitate in isopropanol and 50 mM Na_2_PO_4_ buffer with 0.2% sodium deoxycholate and 0.1% gum Arabic (*w*/*v*) at a ratio of 1:9. After an incubation at room temperature for 1 h, 1 mL of sodium carbonate buffer of 1 M was added to end the reaction. The supernatants were acquired, and the values of OD_410nm_ were determined using a spectrophotometer.

### 2.8. Protease Assay

Protease activities after treatment with different concentrations of turmeric oil were determined by a protocol previously reported by Sun et al. [26]. In brief, 125 μL of 0.3% azocasein was mixed with 75 μL of bacterial supernatant; the reaction systems were incubated at 30 °C for 15 min and then terminated by adding 10% trichloroacetic acid at a volume of 600 μL. The samples were centrifuged to obtain the supernatants, and the values of OD_440nm_ were determined by adding 780 μL of 1 M NaOH to the supernatants.

### 2.9. Biofilm Formation

Turmeric oil was diluted in a 96-well plate to final concentrations of 8 to 64 μg/mL at a volume of 100 μL in each well. The bacterial strain was inoculated in LB medium and cultured to an OD_600nm_ of 1.0. Then, the bacterial culture was 10-fold diluted with fresh LB medium, and 100 μL of it was mixed with the drugs in each well. Bacterial culture without turmeric oil treatment was defined as the positive group, while LB medium served as the negative group. The plate was cultured at 37 °C for 24 h, unattached bacterial cells in each well were discarded, and cells attached to the plate were washed twice with PBS. After air-drying the plate for 30 min, 0.5% crystal violet was added to stain the bacterial cells on the plate. After washing, acetic acid was added to release the crystal violet. The quantity of biofilms was determined by measuring OD_570nm_.

For light microscopic visualization, biofilm was formed in a 24-well plate with glass slides in each well after treatment with certain concentrations of turmeric oil [27]. After incubating for 24 h, the glass slides were washed to remove unattached bacterial cells and were then stained with crystal violet. The glass slides with stained biofilms were then mounted on microscopic slides, and biofilms were imaged by a microscope (Olympus, Tokyo, Japan).

### 2.10. qPCR Assay

Total RNA was isolated from bacterial cells obtained from the hemolysis assay using a commercial RNA extraction kit (Yeasen, Shanghai, China). Reverse transcription was performed to obtain cDNA after determining the RNA concentrations using a micro-volume spectrophotometer. Then, the qPCR reactions were conducted to assess the influence of turmeric oil at the indicated concentrations on the transcription of the *aerA*, *ahyI*, and *ahyR* genes. Primer pairs used for detecting gene expression are shown in Table 1. The 16s rRNA gene was employed as the internal control gene to normalize the levels of expression. The relative transcriptions were calculated using the 2^−ΔΔCt^ method, with CT values obtained from qPCR.

### 2.11. AHLs Determination Assay

AHLs’ production levels of *A. hydrophila* after co-incubation with turmeric oil were investigated according to a previous study [23]. In brief, 1% overnight bacterial cultures of *A. hydrophila* and *C. violaceum* CV026 were streaked in parallel at equal intervals on agar plates with turmeric oil ranging from 8 to 64 μg/mL, respectively. After incubation, the inoculum on the plates was then incubated at 28 °C for 24 h. The bacterial cells of *C. violaceum* CV026 were removed using an aseptic inoculation ring to determine the relative amount of AHLs. Then, the pigments were dissolved in DMSO and analyzed using a spectrophotometer (Unico, Shanghai, China) at OD_585nm_.

### 2.12. Cell Viability Assay

A549 cells (ATCC CCL185) obtained from the American Type Culture Collection (ATCC) were cultured at 37 °C with 5% CO_2_ in a humidified incubator. After digestion using trypsin, the cell density was adjusted to 5 × 10^6^ cells/mL, and 100 μL of cell suspension was added to each well of a 96-well plate. Cells were incubated at 37 °C overnight, and then bacterial supernatants after treatment with 0.22 μm sterile filters were added to each well, while cells without adding bacterial supernatant were defined as the negative group. Cells after different treatments were further incubated for 2 h and then centrifuged. Cells after treatment were washed with PBS three times and were stained with calcein-AM and propidium iodide at 2 μM and 4.5 μM at 37 °C for 15 min according to the instructions supplied by the manufacturer. The images of cells after staining were obtained using a fluorescence microscope (Olympus, Tokyo, Japan). Live cells were stained with calcein-AM, exhibiting green fluorescence, while dead cells were stained with propidium iodide, showing red fluorescence.

### 2.13. Animal Studies

Experimental therapeutics were certified by the Animal Welfare and Research Ethics Committee of our institute and were conducted in accordance with the ARRIVE guidelines 2.0. 90 healthy grass carp (200 ± 10 g) were separated into 3 groups, and 3 biological repeats were contained in each group. *A. hydrophila* XS-91-4-1 was cultured to the mid-log phase, and then centrifugation was performed to obtain bacterial cells. Cells were resuspended to 1.5 × 10^9^ cfu/mL in PBS using McFarland Standards after washing. The fish infection model was set up by an intraperitoneal injection of bacterial suspension at a volume of 200 μL in the positive control group (PG) and the turmeric oil-treated group (TG), while sterile PBS was given to fish in the negative control group (NG) at the same volume. Fish in the turmeric oil-treated group were given turmeric oil using a gavage needle at 30 mg/kg 6 h post-challenge and every 12 h for 3 days, while fish in the PG and NG groups were administered 10% Tween 80. The deaths of fish were recorded for each tank for 8 days. Posterior kidneys of fish in all three groups were removed and fixed in 10% formalin for histopathological analysis.

### 2.14. Statistical Analysis

Statistical significance of in vitro studies was analyzed by the Student’s t-test after determining the normality. The Kaplan–Meier estimates and the log-rank test were employed to determine the significance of the survival rate.

## 3. Results

### 3.1. Chemical Composition of Turmeric Oil

The major compounds in turmeric oil used here were listed in Table 2. According to the results, 35 compounds were detected, ar-turmerone (35.93%) and curlone (14.24%) were the main compounds in turmeric oil.

### 3.2. Impact of Turmeric Oil on Bacterial Growth

**Figure 1:**
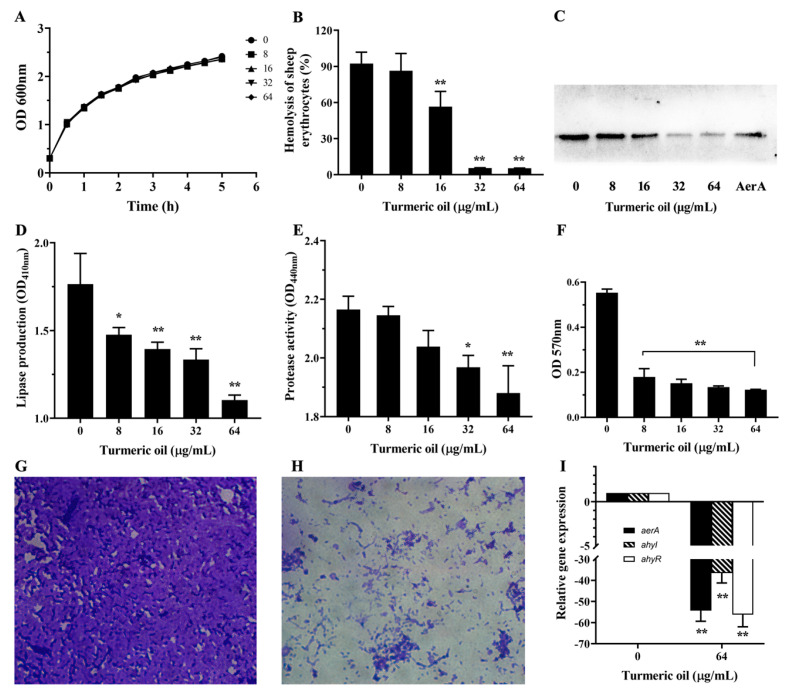
Turmeric oil reduced the phenotypes mediated by quorum sensing. (**A**) Growth trends of *A. hydrophila* XS-91-4-1 with turmeric oil at 0, 8, 16, 32, and 64 μg/mL. (**B**) Turmeric oil dose-dependently inhibited the hemolysis of bacterial supernatants. (**C**) Turmeric oil reduced the quantity of aerolysin in bacterial supernatants. (**D**) Turmeric oil affects the production of lipase. (**E**) Inhibitory effect of turmeric oil against protease activity. (**F**) Inhibitory effect of turmeric oil on biofilm formation. (**G**,**H**) Biofilm formation on glass slides; (**G**) Turmeric oil-free group; (**H**), 64 μg/mL turmeric oil-treated group. (**I**) Turmeric oil suppressed the transcriptions of target genes. The data shown in (**A**) were the mean values of three independent experiments, and the data in (**B**–**F**,**I**) were the mean values with standard deviations (SD) of three independent experiments. * indicated 0.01 < *p* < 0.05, and ** indicated *p* < 0.01, compared with the turmeric oil-free group.

The MIC of turmeric oil against the tested bacterial strain was 256 μg/mL, indicating that turmeric oil had little anti-bacterial activity. Subsequently, the results of the growth curve assay meant that the turmeric oil, ranging from 8 to 64 μg/mL, had no effect on bacterial growth (Figure 1A). Furthermore, DMSO did not impact bacterial growth by adding DMSO in the turmeric oil-free group (Figure 1A). Therefore, these results demonstrate that turmeric oil at our experimental concentrations had no selective pressure on *A. hydrophila* XS-91-4-1.

### 3.3. Turmeric Oil Decreased Hemolysis by Affecting Aerolysin Secretion

Though turmeric oil could hardly affect the growth of *A. hydrophila* at 0 to 64 μg/mL, the hemolytic activities of cell-free supernatants were dose-dependently inhibited after wco-incubation with turmeric oil (Figure 1B). The relative hemolytic activity was reduced to 86.35 ± 14.42, 56.53 ± 12.65, 5.50 ± 0.19, and 5.30 ± 0.13% with turmeric oil at 8, 16, 32, and 64 μg/mL, while the turmeric oil-free group was 92.43 ± 9.45% (Figure 1B). When the concentration of turmeric oil reached 8 μg/mL, turmeric oil could significantly inhibit the hemolysis of bacterial supernatants (Figure 1B). These observations revealed that the production or activity of aerolysin was inhibited by the addition of turmeric oil. Therefore, the quantity of aerolysin was further determined using bacterial supernatants by Western blot. As expected, the amount of aerolysin decreased with the increasing concentrations of turmeric oil (Figure 1C). Taken together, these results meant that turmeric oil could suppress the secretion of aerolysin in a dose-dependent manner.

### 3.4. Turmeric Oil Inhibited Lipase and Protease Production

The quantity of lipase and protease secreted by *A. hydrophila* after co-incubation with turmeric oil was analyzed using bacterial supernatants. As shown in Figure 1D, turmeric oil inhibited the production of lipase in a dose-dependent manner. Turmeric oil could remarkably reduce the production of lipase at concentrations of 8 μg/mL and above. Moreover, similar results were achieved in protease production (Figure 1E). Turmeric oil could remarkably reduce the production of protease at concentrations higher than 16 and 32 μg/mL (Figure 1E).

### 3.5. Turmeric Oil Influenced Biofilm Formation

Biofilm can reduce the therapeutic effect of antibiotics and may be a source of iterative and continual infections. Thus, we detected the biofilm formation of *A. hydrophila* after co-incubation with turmeric oil at certain concentrations. Biofilm formation was significantly inhibited with turmeric oil of 8, 16, and 32 μg/mL (Figure 1F). The relative inhibitory rate was 77.89 ± 0.33% with 64 μg/mL turmeric oil. Moreover, the structure of biofilm on glass slides was analyzed after treatment with a certain concentration of turmeric oil. As shown in Figure 1G, a dense biofilm was observed without treatment with turmeric oil, while a visible decrease in biofilm was observed in the 64 μg/mL turmeric oil-treated group (Figure 1H).

### 3.6. Turmeric Oil Decreased Related Genes Relative Expression

The inhibition of aerolysin, lipase, and protease production and biofilm formation revealed that turmeric oil might be an inhibitor of QS. Therefore, the transcriptions of genes involved in aerolysin expression (*aerA*) and QS (*ahyI* and *ahyR*) were detected by qPCR, respectively. The results showed that turmeric oil at 64 μg/mL could significantly reduce the transcription of *aerA*, *ahyI*, and *ahyR*; a 54-fold, 36-fold, and 56-fold reduction was observed for each gene compared with the turmeric oil-free group (Figure 1I). The result demonstrated that turmeric oil could disrupt the function of bacterial QS and result in a decrease in bacterial virulence regulated by QS.

### 3.7. Turmeric Oil Decreased AHLs Production

**Figure 2:**
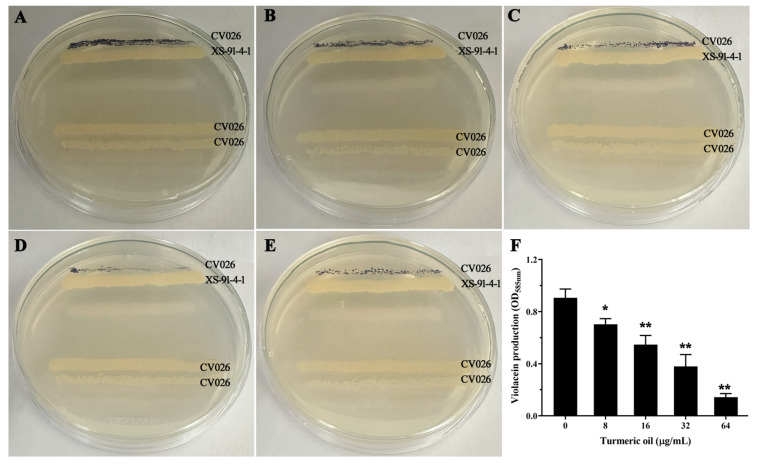
Turmeric affected the amount of AHLs produced by *A. hydrophila*. (**A**–**E**) Streak assays for AHLs production in *A. hydrophila* co-cultured with turmeric oil using *C. violaceum* CV026; (**A**) Turmeric oil free group; (**B**) 8 μg/mL turmeric oil-treated group; (**C**) 16 μg/mL turmeric oil-treated group; (**D**) 32 μg/mL turmeric oil-treated group; (**E**) 32 μg/mL turmeric oil-treated group. (**F**) Violacein production of *C. violaceum* CV026 co-incubated with *A. hydrophila* and turmeric oil at certain concentrations. * indicated 0.01 < *p* < 0.05, and ** indicated *p* < 0.01.

To analyze the role of turmeric oil on the production of AHLs, we employed *C. violaceum* CV026 as a reporter strain. *C. violaceum* CV026 could form purple pigment when co-incubated with AHLs produced by *A. hydrophila* XS-91-4-1 (Figure 2A). But the pigment was decreased with the increasing concentrations of turmeric oil (Figure 2B–E), indicating that turmeric oil could affect the amount of AHLs in a dose-dependent manner. Moreover, the relative amount of AHLs was determined by evaluating the absorption of the violacein. As expected, the production of AHLs was significantly decreased by turmeric oil at concentrations of 8, 16, and 32 μg/mL (Figure 2F). Taken together, the results demonstrated that the decrease of AHLs led to the down-regulation of QS and virulence factors.

### 3.8. Cell Viability Results

**Figure 3:**
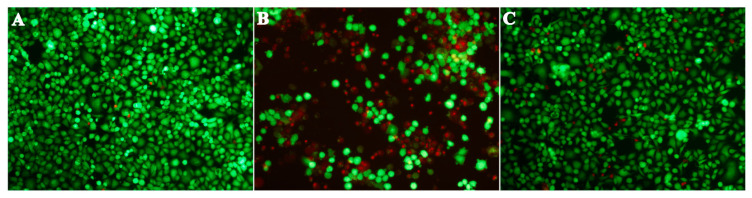
Viability of A549 cells after treatment with bacterial supernatants. Cells were labeled by live/dead staining regents, live cells were green, while dead cells were red. (**A**) Untreated cells; (**B**) Cells treated with turmeric oil-free supernatant; (**C**) Cells treated with bacterial supernatant plus turmeric oil at 128 μg/mL.

It is known that bacterial supernatant containing aerolysin can cause cell injury in a number of mammalian cells. Thus, A549 cells were used to analyze the cell injuries mediated by aerolysin in bacterial supernatants. As shown in Figure 3A, A549 cells without supernatant treatment showed green fluorescence, suggesting that the cells were alive, while cells co-incubated with turmeric oil-free supernatant showed a large amount of red fluorescence (Figure 3B), indicating that A549 cells were dead after treatment with bacterial supernatant. A549 cells after treatment with bacterial supernatant co-cultured with 64 μg/mL turmeric oil showed a visible decrease in red fluorescence (Figure 3C), indicating that dead cells were decreased.

### 3.9. Turmeric Oil Protected Grass Carps from A. hydrophila-Induced Mortality

**Figure 4:**
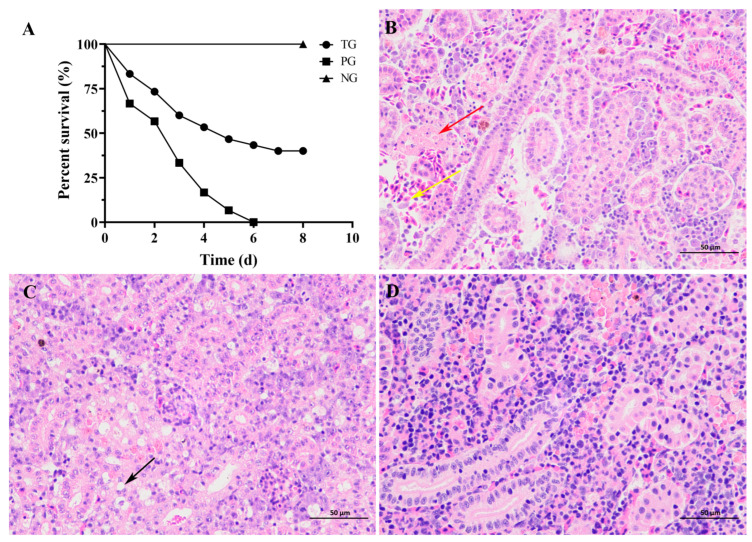
Turmeric oil reduced the mortality and kidney injury of grass carps challenged with *A. hydrophila*. (**A**) Survival rate of fish treated with or without turmeric oil post *A. hydrophila* infection. (**B**–**D**) Histopathological changes of posterior kidneys in different groups (×400). (**B**) Histological observation of the kidney from fish challenged with *A. hydrophila* only. Yellow arrow: renal interstitial congestion; red arrow: the renal tubular epithelial cells degeneration and necrosis, shedding into the tubular lumen. (**C**) Histological observation of the kidney from fish infected with *A. hydrophila* and treated with turmeric oil. Black arrow: the renal tubular epithelial cells showed vacuolar degeneration, and no obvious necrosis was observed. (**D**) Histological observation of the kidney from a fish without any treatment.

The in vitro results suggested that the turmeric oil might have potent therapeutic effects on fish infected with *A. hydrophila*. Thus, an *A. hydrophila* infection model was established to investigate the in vivo protection of turmeric oil. Fish infected with *A. hydrophila* showed typical symptoms, including lethargy, unresponsiveness, and a distended abdomen. Deaths were observed in fish in PG and TG 24 h post-infection (Figure 4A). All fish in PG were dead in 6 days, while 40% were alive in TG (Figure 4A). Turmeric oil could remarkably decrease the mortality mediated by *A. hydrophila* infection compared with TG. Moreover, renal injury was determined after being challenged by *A. hydrophila*. The kidney in the positive control group showed obvious lesions, including renal interstitial congestion (yellow arrow), renal tubular epithelial cells degeneration and necrosis, and shedding into the tubular lumen (red arrow, Figure 4B). In contrast, the kidneys in the turmeric oil-treated group showed vacuolar degeneration in the renal tubular epithelial cells; no obvious necrosis was observed (black arrow, Figure 4C), compared with the kidney from the fish without any treatment (Figure 4D). Taken together, turmeric oil treatment could reduce the mortality and renal lesions caused by *A. hydrophila*.

## 4. Discussion

The rapid growth of the global population and economy has increased the demand for high-protein food sources. In this context, aquaculture has become an integral component in the food supply chain [28]. The increasing demand for aquatic products has resulted in higher animal densities in limited spaces compared to previous decades. However, the occurrence of infectious diseases has become a reason for economic loss [29]. Furthermore, common practices, including intensive aquaculture operations and infectious disease control, might favor evolution toward higher pathogen virulence, as theory predicts [30]. Thus, controlling the emergence and spread of pathogens is crucial for the high-quality development of aquaculture. Antibiotics are widely used to control bacterial diseases in aquaculture, but antibiotic resistance has limited their applications. Moreover, antibiotic resistance brought serious consequences for the health of both animals and humans involved in aquaculture [31].

Chinese traditional medicine (CTM) has been utilized for thousands of years to deal with diseases in both humans and animals. In contrast to antibiotics used in aquaculture, CTMs derived from plants and their extracts have several advantages, including affordability, ease of access, minimal toxicity, and few adverse effects. These characteristics make CTMs an appealing alternative for dealing with diseases in fishery practices [32]. Several studies have demonstrated the inhibitory effects of turmeric and its extracts in preventing *A. hydrophila* infection or as QS inhibitors. Abdel-Tawwab M and Abbass F E demonstrated that turmeric powder could prevent *A. hydrophila* infection by improving growth performance and innate immunity [33]. Azizah N and Aji R showed that turmeric rhizome infusion, with multi-bioactive compounds, had anti-*A. hydrophila* activity in vitro, which may be a potent drug in dealing with *A. hydrophila* infections [34]. Moreover, studies have demonstrated that curcumin is a natural QS inhibitor of *Bacillus subtilis*, *Pseudomonas aeruginosa* (*P. aeruginosa*), *Escherichia coli*, *Proteus mirabilis*, and *Serratia marcescens* [35,36]. However, the main compound of turmeric is curcumin, which is absolutely different from turmeric oil. According to our findings, aR-turmerone was the main compound in turmeric oil (Table 2). Therefore, the inhibitory effects of turmeric oil against *A. hydrophila* infection need to be clarified. Essential oils isolated from CTMs were mixtures of natural aromatic volatile oils and have been widely used in traditional and complementary medicines with various biological activities [37]. Chowdhury H et al. demonstrated that the components of *Cymbopogon flexuosus* oil could strongly bind with DNA gyrase-B of *A. hydrophila* and exhibited anti-bacterial activity against two oxytetracycline-resistant *A. hydrophila* [38]. Zhong et al. showed that *Satsuma mandarin* oil can affect the growth of *A. hydrophila* by interfering with extracellular membrane permeability [39]. Turmeric oil with ar-turmerone as the major bioactive compound is reported to have anti-bacterial activity against a variety of bacteria. Jayaprakasha et al. demonstrated that turmeric oil has antifungal activities with a broad spectrum, which significantly inhibited the growth of *Aspergillus flavus*, *Aspergillus parasiticus*, *Fusarium moniliforme*, and *Penicillium digitatum* [40]. Kumar V et al. found that turmeric oil could enhance immunity and induce resistance of fish against *Ichthyophthirius multifiliis* and *A. hydrophila* co-infection [21]. Whether turmeric oil could affect the growth or virulence of *A. hydrophila* is not yet determined. Jamuna Bai A and Ravishankar Rai Vittal showed that essential oils, including turmeric oil, are QS inhibitors of *P. aeruginosa* in food systems; however, *A. hydrophila* was not studied [41]. Taking the previous findings together, it is necessary to investigate the anti-infective effect of turmeric oil against aquatic pathogens. In the present study, we found that turmeric oil had little anti-bacterial activity, but turmeric oil could decrease the secretion of several virulence factors regulated by QS at 8 to 64 μg/mL. Moreover, Histopathological results showed that fish after treatment with turmeric oil only exhibited vacuolization, compared with the tubular necrosis of the positive control group. The findings were similar to those reported by Chen et al. [42]. These findings demonstrated that turmeric oil was a potent modulator of bacterial virulence, which could be used as an alternative against *A. hydrophila* infections. Moreover, the results could help understand the mechanism of turmeric oil against *Ichthyophthirius multifiliis* and *A. hydrophila* co-infection, as described by V. Kumar [21].

Although the MICs of essential oils were much higher than those of natural compounds, their anti-infective effects were determined by anti-virulence strategies. Luo et al. demonstrated that lemon oil could inhibit the glycolytic pathway of *Streptococcus mutans*, resulting in a reduction of LDH expression and activity at sub-inhibitory concentrations, which clarified the anti-caries activity of lemon essential oil [43]. Huang found that zedoary turmeric oil could inhibit the growth of *Listeria monocytogenes* and *Staphylococcus aureus* at concentrations ranging from 1 to 2 mg/mL. Moreover, zedoary turmeric oil at sub-inhibitory concentrations could decrease the production of exotoxin proteins in both bacteria; the findings might further the progress of novel anti-infective drugs [44]. Li et al. demonstrated that neem oil without anti-bacterial activity could reduce the pathogenicity of *A. hydrophila* by disrupting QS and biofilm formation at concentrations ranging from 16 to 128 μg/mL. The findings indicated that essential oils isolated from CTMs could be potent agents for dealing with bacterial infections in aquaculture. Moreover, Jamuna Bai A and Ravishankar Rai Vittal found that seven kinds of essential oils, including turmeric oil, could decrease the QS-regulated virulence factors of *P. aeruginosa* PA01; the findings revealed that turmeric oil might be a QS inhibitor of *A. hydrophila* [41]. Thus, studying the anti-infection effect of turmeric oil is of great significance for promoting the health of aquatic animals. However, the dosage of turmeric oil needs to be further optimized to achieve the best therapeutic effect in dealing with bacterial infections. In addition, it is important to prepare a suitable dosage form to improve the bioavailability of turmeric oil in fish. Therefore, there is still a long way to go before turmeric oil can be used as a legal therapeutic medicine in aquaculture. The study was the first to determine the inhibitory effect of turmeric oil against *A. hydrophila* infection by interfering with the QS. The study not only provided a potent candidate for aquaculture dealing with *A. hydrophila* infection but also partly explained the anti-infective effect of turmeric. It is important to note that a previous study has demonstrated that the compositions and expression levels of bacterial virulence factors may result in differences in pathogenicity among different strains [45]. Therefore, the therapeutic effect of turmeric oil may be somewhat different from that reported in this study. The inhibitory effect of turmeric oil against QS depended on the compounds in the oil. Moreover, studies have demonstrated that essential oils show stronger anti-bacterial activities than compounds alone isolated from essential oils, indicating that the compounds in essential oils have synergistic effects to achieve maximum efficacy [46,47]. Our results showed that ar-turmerone (35.93%) and curlone (14.24%) were the main components of turmeric oil; these findings were similar to previous studies [48,49]. However, the contents of compounds in essential oils are easily affected by several factors, such as origin, processing methods, and environment [50]. Therefore, it is critical to standardize the contents of essential oils for their further applications. Our study focused on screening QS inhibitors from essential oils and determining the mechanism of turmeric oil; the inhibitory effects of compounds in essential oils were not determined, which might limit further application of turmeric oil due to the variability in composition and content of different sources.

## 5. Conclusions

The study clarified the inhibitory mechanism of turmeric oil against virulence factors regulated by QS and determined the protective effect of turmeric on fish challenged with *A. hydrophila*. These findings partly clarified the mechanism of turmeric against bacterial infections and provided a novel approach in exploring anti-infective drugs in aquaculture.

## Figures and Tables

**Table 1:** Primer pairs used in qPCR.

Primer	Sequence	PCR Amplicon (bp)
*aerA*-F *aerA*-R	TCTACCACCACCTCCCTGTC GACGAAGGTGTGGTTCCAGT	218
*ahyI*-F *ahyI*-R	GTCAGCTCCCACACGTCGTT GGGATGTGGAATCCCACCGT	202
*ahyR*-F *ahyR*-R	TTTACGGGTGACCTGATTGAG CCTGGATGTCCAACTACATCTT	206
16S rRNA-F 16S rRNA-R	TAATACCGCATACGCCCTAC ACCGTGTCTCAGTTCCAGTG	164

**Table 2:** Components presented in turmeric oil after analyzed by GC-MS.

No.	Compound Name	Retention Time (min)	Peak Area (%)
1	α-pinene	6.532	0.03
2	α-phellandrene	7.282	0.61
3	3-carene	7.342	0.04
4	curlone	12.337	14.24
5	p-cymene	7.485	0.40
6	eucalyptol	7.557	0.99
7	cyclohexene	8.069	0.54
8	7-epi-sesquithujene	10.465	0.13
9	naphthalene	10.537	0.13
10	β-bisabolene	11.145	1.83
11	α-santalene	10.608	0.15
12	caryophyllene	10.668	0.91
13	(+)-cycloisolongifol-5-ol	10.799	0.68
14	methyl DHA	10.847	0.08
15	amorphadiene	10.894	0.50
16	α-curcumene	10.990	4.56
17	1-zingiberene	11.063	6.22
18	2-ethyl-m-xylene	11.610	2.69
19	Di-epi-cedrene-(I)	11.884	2.15
20	aR-turmerone	12.122	35.93
21	(Z)-γ-atlantone	12.265	0.62
22	α-terpinene	7.402	0.05
23	(6r, 7r)-bisabolone	12.587	2.37
24	tumerone	12.659	1.14
25	(E)-atlantone	12.730	3.51
26	β-sesquiphellandrene	11.252	5.95
27	β-santalol	12.957	1.06
28	Retinal	13.088	0.55
29	6-epi-shyobunol	13.243	0.46
30	ergocalciferol	13.338	0.81
31	isolongifolol	13.445	0.24
32	n-hexadecanoic acid	13.600	1.29
33	patchouli alcohol	13.875	0.75
34	(E)-γ-bisabolene	11.300	0.49
35	linoleic acid	14.483	3.59

## Data Availability

The data that support the findings of this study are available from the corresponding author upon reasonable request.
